# Assessment of dropout rates in the preclinical years and contributing factors: a study on one Thai medical school

**DOI:** 10.1186/s12909-022-03527-z

**Published:** 2022-06-16

**Authors:** Sorawit Wainipitapong, Mayteewat Chiddaycha

**Affiliations:** 1 Department of Psychiatry, Faculty of Medicine, Chulalongkorn University and King Chulalongkorn Memorial Hospital, the Thai Red Cross Society, Bangkok, Thailand; 2 Center of Excellence in Transgender Health, Faculty of Medicine, Chulalongkorn University, Bangkok, Thailand

**Keywords:** Dropout, Medical education, Medical students, Examination, Mental problems, Mental, Mental health, Mental health problems, Admission program, Thailand

## Abstract

**Background:**

The highest dropout rate for medical students is during the preclinical years of education. Several studies have reported possible contributing factors, however, studies regarding the dropout rate from medical education among Thai medical students and its associated factors are still lacking. This study aimed to identify the prevalence of dropout from medical education within the preclinical period and its associated factors among Thai medical students.

**Methods:**

We collected data from preclinical medical students who entered one medical school in Bangkok, Thailand, between 2017–2019. Demographic data, admission program, pre-admission mental health status assessed by the Thai Mental Health Indicator 66, mental health records from the faculty-based counseling services, and academic achievement were extracted. Data were reported in a descriptive fashion. We analyzed the association between dropout and these factors by robust Poisson regression.

**Results:**

In total, 914 students were recruited. Dropout was only 1.5%, which was lower than the previous studies. Regression analysis showed a significant association between dropout and mental health problems [Prevalence ratio (PR) 58.20, 95%CI 13.72- 246.95] but not admission program [PR 0.32, 95%CI 0.09 – 1.16] or failing examinations [PR 0.59, 95%CI 0.18 – 1.90]. However, other contributing factors such as financial status, family problems, medical illness, and students’ motivation, were not evaluated in this study.

**Conclusions:**

Mental health problems during medical education were associated with dropout after adjusting for other confounding variables. Further longitudinal studies are needed to identify the impacts of academic failure on dropout in higher clinical years.

## Background

Medical education ultimately requires comprehensive growth in a wide range of dimensions to serve the needs of patients and the society, shaped by the attitudes towards physicians in each cultural context [1]. Medical students with greater developmental maturity that comes with increasing age may be better prepared for medical education [2]. Higher exposure to humanities is reported to reduce burnout during medical education [3]. However, these protective factors against stress during medical school demand more time for personal growth, which is sadly restricted in the context of Thailand’s educational system.

In contrast to some western countries, entry to medical education in Thailand begins immediately after a high school degree. In comparison with other countries where students enter medical school in their early 20 s [2], Thai students are required to commit to the long and hard process of becoming a physician at approximately eighteen years of age. The competition for university admission is one leading cause of significant stress among Thai high school students [4]. Transitioning into medical education, however, may be an even more stressful event.

Medical students who carry such stress developed burnout and intention to leave medical education [5]. Dropout not only causes a waste of time of individual medical student but also dissipation of taxpayers’ funding or effort of educators [6]. In addition, psychological, personal, and financial consequences of dropout should also be recognized, particularly in the specific sociocultural context of each country, because of differences in rate of dropout or medical education curriculum [7, 8]. The dropout from medical school consequently received more academic attention in order to identify causes and reduce its rate for preventing unfavorable sequelae, individually and economically.

Factors leading to dropout may be increased demands on academic achievement, stress, and mental health problems. Previous studies have found an association between academic problems and stress [9], while high academic achievement was associated with less sleep and poor sleep quality. Sleep problems, in turn, were associated with depression, anxiety, and stress [10, 11]. These findings show that the interplay between academic achievement, sleep, and mental health problems is complex and may require further study. Academic failure is considered to be a major predictor of dropout in medical education, while the influence of mental health problems has not been thoroughly investigated [12]. Serious thoughts of dropping out are associated with burnout and depressive symptoms [13] but there is no significant association between dropout and either past or current mental health problems [14]. However, poor mental health could predict dropouts in some educational levels and genders [15]. Negative experiences during clinical placement, which might be related to mental issues, could lead to dropout in nursing education, whereas academic difficulty played a major role in early dropout students [16]. Within the initial years of medical education, which mental problems are common causes of academic struggles [17], dropout was also associated with entry qualifications, such as admission test scores and different admission programs [18, 19].

There are two main admission programs in Thai medical education. The Consortium of Thai Medical Schools (COTMES) organizes the national admission examination for all applicants with a high school degree or higher. The COTMES program is the ordinary admission track for most Thai high school students. Another program aims to increase the number of rural doctors needed in some distant provinces. The eligibility of applicants is limited by the region in which they live. Differences in admission scores but not a grade point average (GPA) during medical school are found regarding the specific track they choose in this program [20]. Compared to COTMES program, students in this program have poorer academic performance during the preclinical years but, conversely, perform comparably or even better at their license examination in later academic years [21]. However, to the best of our knowledge, the type of admission program has not been studied for its association with dropout from Thai medical education.

The preclinical years pose different impacts on students compared to the clinical years. Mental health problems, commonly including depression, anxiety, and suicidal ideation, reach their peak incidence during the preclinical years [22–24]. The impact of mental health on dropout should therefore be a focus of research in preclinical students, along with other factors which may also contribute to this problem. Additionally, each country is unique in its demographic profile, educational policy, and societal attitudes toward the medical profession. We aim to examine the prevalence of dropout from medical education within the preclinical period and its associated factors in one Thai medical school under the context of Thailand, where medical students are school leavers and have distinct sociocultural considerations.

## Methods

### Study design and population

We collected the data from medical students who entered one medical school between 2017–2019 in Bangkok, Thailand. Prior to admission, all students were divided into different groups according to the program they applied for and received a mental health screening by a self-reported questionnaire. At the end of the 2019 academic year, the records of their academic achievement during their first to third year of medical education, including failing examinations and dropout, were extracted. Mental health data after admission were collected from the faculty-based counseling service, which was provided by certified psychotherapists and psychiatrists. Written informed consent was obtained from all students. We collected the data from all medical students throughout the study period. Thus, neither sampling nor sample size calculation was done. This study was approved by the Institutional Board Review of the Faculty of Medicine, Chulalongkorn University, Bangkok, Thailand (IRB No. 065/2020).

Most students were school leavers and applied to the program organized by COTMES. This admission route is open for applicants from all around the world. Meanwhile, eligibility for alternative programs to increase rural doctors is limited to the applicant whom resides in regions where doctors are considered lacking [25]. We classified all students into COTMES and non-COTMES groups according to their programs. Demographic data including sex, year of study, and hometown were also collected.

### Academic achievement

Poor academic achievement of the students was assessed by two established variables, failing examinations and dropout, which were categorized separately for analysis. According to the academic grading in Thai medical school, students whose score were lower than the minimal passing level would receive an F in their transcripts. Those with at least one F grade in their recorded data were categorized as ‘failing examinations’ in our study. We used ‘dropout group’ to describe medical students who had decided to abandon the medical education because of the decision to change their career or poor academic performance, including retirement following incompetent cumulative GPA or educational difficulties. Other causes of dropout such as resignation to study abroad or hold a scholarship, were excluded. All data were provided by the Division of Academic Affairs.

### Mental health

Approximately three months before the commencement of medical education, the Thai Mental Health Indicator 66 was used to evaluate the pre-admission mental health status of all medical students in our study [26]. The questionnaire consisted of 66 self-report items and was validated for use among Thai people aged between 15 to 60 years. Four domains including mental status, mental capacity, mental quality, and supporting were measured. Each domain reliability is 0.86, 0.83, 0.77, and 0.80, respectively. Students screened positive by the test were classified as ‘poor mental health’ in our study. The questionnaire can be publicly downloaded from the website of the Department of Mental Health, Ministry of Public Health, Thailand (http://www.dmh.go.th/test/qtnew/). With all clients’ consent, we extracted mental health records from the faculty-based counseling service, where medical students who needed mental health evaluation and support requested an appointment by themselves or referred by their advisors. All students at this center would be assessed by attending psychiatrists for precise diagnoses and proper bio-psychological interventions. We collected the psychiatric diagnoses of all clients who met inclusion criteria for this study throughout our study period.

### Statistical analysis

We used STATA 16 (John Wiley & Sons, Inc.) for data analysis. Descriptive statistics were used to report demographic data, mental health, and academic achievement. Categorical variables were presented as counts and percentages. We used Fisher’s exact test to determine the difference among categorical data. Multivariable regression was used to explore association between dropout and associated factors. A *p value* of < 0.05 is considered statistically significant. Since all medical students within the study period were recruited, sampling and sample size calculation were not done.

## Results

A total of 955 medical students were admitted to one medical school located in Bangkok, Thailand, from 2017 to 2019. Students have spent one to three years in medical education depending on their academic year of entry. We excluded 41 students who quitted before starting their first semester. Ten students who resigned to attain overseas scholarship were also excluded and not counted in the dropout group. In total, 914 medical students were enrolled in this study. Demographic profile, mental health problems, and academic achievement were shown in Table 1.

**Table 1:** Demographic data and univariate analysis of factors among dropout and not dropout students

Factors	Total (*N* = 914)		Not dropout (*N* = 900)		Dropout (*N* = 14)		*p value*
	*n*	%	*n*	%	*n*	%	
Sex							0.592
Female	432	47.3	424	47.1	8	57.1	
Male	482	52.7	476	52.9	6	42.9	
Year of study							0.007*
1^st^ Year	307	33.6	307	34.1	0	0	
2^nd^ Year	319	34.9	313	34.8	6	57.1	
3^rd^ Year	288	31.5	280	31.1	8	42.9	
Admission program^a^							0.008*
COTMES	594	65.0	590	65.6	4	28.6	
Non-COTMES	320	35.0	310	34.4	10	71.4	
Hometown							0.177
Bangkok and metropolitan	506	55.4	501	55.7	5	35.7	
Others	408	44.6	399	44.3	9	64.3	
Pre-admission mental health							0.365
Poor	29	3.2	28	3.1	1	7.1	
Normal	885	96.8	872	96.9	13	92.9	
Mental health problem							< 0.001*
Yes	85	9.3	73	8.1	12	85.7	
No	829	90.7	827	91.9	2	14.3	
Psychiatric Diagnosis							
Depressive disorder	24	28.2	17	1.9	7	50.0	< 0.001*
Adjustment disorder	24	28.2	21	2.3	3	21.4	0.005*
Anxiety disorder	20	23.5	19	2.1	1	7.1	0.268
OCD	5	5.9	5	0.56	0	0	1.000
Bipolar disorder	2	2.4	2	0.22	0	0	1.000
Learning problem	2	2.4	2	0.2	0	0	1.000
ADHD	3	3.5	3	0.3	0	0	1.000
Insomnia	2	2.4	2	0.2	0	0	1.000
Others	3	3.5	2	0.2	1	7.1	0.045*
Failing examination^b^							0.044*
Yes	92	10.1	88	9.8	4	28.6	
No	822	89.9	812	90.2	10	71.4	

^*^p < 0.05

^a^ Admission program was grouped into COTMES program students which applied nationwide and other programs that restricted application from specific rural provinces

^b^ Failing examination was defined by receiving at least one academic F grade

*Abbreviations;* *COTMES* the Consortium of Thai Medical Schools, *ADHD* Attention deficit/hyperactivity disorder, *OCD* Obsessive compulsive disorder

The mean age at admission was eighteen years old, with male medical students being slightly greater in number (52.7%). The majority applied through the COTMES program (65.0%) and their hometowns were located in Bangkok and metropolitan areas (55.4%). The number of medical students in each year was approximately comparable (288 to 319 students). None of the first-year students drop out from medical education. Pre-admission mental health status was remarkably normal (96.8%), and 85 students (9.3%) sought mental health treatment during studying. Among those with such issues, the most frequent diagnoses were depressive disorder (28.2%), adjustment disorder (28.2%), and anxiety disorder (23.5%). In total, 92 students (10.1%) received at least one academic F grade.

During three consecutive academic years, fourteen students (1.5%) had dropped out. The results from univariate analysis revealed no significant association between dropout and factors such as sex, hometown, and pre-admission mental health. However, a significant association with academic year (*p* = 0.007), admission program (*p* = 0.008), mental health problems (*p* < 0.001), and failing examinations (*p* = 0.044) were found. (Table 1).

Multivariable regression was used for further investigation as shown in Table 2. After adjusting for sex, admission program, hometown, pre-admission mental health, mental health problems during the study, and failing examinations, dropout was significantly associated with having mental health problems during medical education (*p* < 0.001) with a prevalence ratio of 58.20 (95% CI 13.72–246.95). Meanwhile, there was no association between dropout and students’ admission program (*p* = 0.375) or failing examinations (*p* = 0.083) in adjusted model.

**Table 2:** Multivariable regression between dropout and associated factors

Variables	Prevalence Ratio	95% confidence interval		*p value*
		lower	upper	
Male	0.66	0.23	1.86	0.428
COTMES program	0.32	0.09	1.16	0.083
Hometown outside Bangkok and metropolitan	0.84	0.27	2.65	0.765
Poor pre-admission mental health	2.36	0.58	9.55	0.228
Mental health problems	58.20	13.72	246.95	< 0.001*
Failing examination	0.59	0.18	1.90	0.375

^*^p < 0.05* Abbreviation; COTMES* The Consortium of Thai Medical Schools

## Discussion

The dropout rate of medical students at this medical school has shown to be lower than a different institute from previous study [27]. However, the forementioned study measured ‘dropout thought’, while our study measured the actual dropout number, which may be understandably lower. When compared to the reported dropout and medical school attrition rate from the United States and the United Kingdom, our study revealed a significantly lower rate of dropout [28, 29]. Meanwhile, the dropout rate from Canadian medical education is also lessened and comparable to our study, which could possibly be explained by academic guidance and mental health supports [29]. According to this university’s educational policy, students whose GPA are lower than 2.0 were at risk for retirement. Those students will be required to achieve a high enough GPA in order to pass the probation within designated periods of an academic semester. Unless the requirement is fulfilled, they would be compelled to drop out by themselves or receive retirement, which would leave an undesirable records for most students. The faculty staff may offer a ‘minimal subject registration’ strategy to purposively maintain students’ GPA or suggest they take a temporary leave from the program. These strategies may have helped to prevent dropout among medical students who were struggling academically. Consequently, failing examinations were then not associated with dropout. Some students received multiple academic F grades and still did not abandon medical education. Academic support could prolong their time in the program or even prevent dropout for these students. A longer follow-up period into their clinical years of medical education may reveal more long-term outcomes of students with poor academic performance.

Pre-admission mental health shown no associated with dropout. This finding was consistent with the previous study [14]. One study from Thailand reported the link between pre-admission mental health and mental health service used after entering medical education, nevertheless dropout was not mentioned [30]. Pre-admission mental health had not received much attention in previous literatures, in contrast to pre-admission academic achievement which is widely studied and tended to be a protective factor against dropout or academic failure [31, 32]. Age could be an important confounding factor because the age of Thai medical students is comparably younger than in some countries. Adolescents and adults have different psychological problems, which could differently influence pre-admission mental health [33]. Thai medical students are mostly school leavers (those entering medical education right after finishing their high school). The range of dropout or attrition rate from other Asian countries where medical students who were school leavers, such as Saudi Arabia, Pakistan and China, varied between 3.8–13.7% [34, 35]. However, updated study on dropout among medical students from several Asian countries are still lacking. More research on the association between dropout and pre-admission mental health along with academic achievement, specifically in non-western context, is necessary.

Mental health during medical education is linked to dropout during the preclinical years, as previous studies have reported [12, 36, 37]. Poor mental health is related to the intention to leave the education [38], especially in students who suffered from more severe symptoms and diagnosed with psychiatric disorders. According to the diagnostic criteria, psychiatric disorder diagnoses are accompanied by impairment in functioning, which for a student, would usually implicate an impairment in studying [39]. It is therefore unsurprising that the dropout rate would be higher among students with poor mental health and academic failure. Lower level of mental health care seeking was also reported and students might access psychological support after the coming of crises [40]. Delay in adequate psychiatric treatment is common globally [41] and potentially leads to dropout. One important factor includes negative attitude or stigma, which is frequent among medical students [42]. Several studies found stigma to be a strong barrier to reach mental health services not only medical students but also all general mental health service users [43–47]. Stigma reduction is challenging, for the most effective way is still disputable [48, 49]. Thus, medical students’ dropout is not easy to handle and mental health burden, as well as its stigmatization, plays a major role regarding dropout reduction strategy.

However, psychological problems alone cannot wholly explain the causes of dropout [16]. The complex etiology of dropout should be viewed as multifactorial, with mental health as one of many variables. Psychological and spiritual issues, including peer support, gratifying parents, and passion for being a doctor, are also positive factors for maintaining students in Thai medical education [27]. Apart from dropout, aforementioned factors also affect mental health in bidirectional mechanism. Financial stress, poor family relationship and not living with both parents are positively associated with psychiatric problems [50, 51]. While positive motivational attitudes and higher parents’ educational attainment could protect against mental health problems [52, 53], and indirectly prevent dropout. Interestingly, our participants from the dropout group were found rather small. None of them reported having a serious medical morbidity that link to dropout. Future qualitative research would be helpful to examine the complicated etiology of dropout. Even though a strong association between psychological problems and academic achievement was reported [54], our study found an association between dropout and having mental problems but not failing examinations. Hence, medical students receiving unsatisfactory examination results were encouraged to be hopeful and continue putting in their best efforts during medical education, together with academic assistance from the faculty staff.

The type of admission program was not significantly associated with dropout in the adjusted model. The type of admission examination was reported to be a strong predictor of dropout [31] in other countries, however, the implications of differing routes of admission in different countries would likely vary according to the nature of respective admission systems. Because of the popularity of this university, a number of Thai high school students chose to enter this medical school regardless of the admission program. Students from the non-COTMES program at this medical school had relatively high pre-admission academic performance, although students from the non-COTMES program are usually less competitive in terms of pre-admission academic achievement. This could explain why there was a significant association in univariate analysis but not in the adjusted model since the academic performance during medical education of COTMES and non-COTMES medical students is comparable [21].

Our study found no association between dropout and sex, which correlated with the inconclusive results from previous studies [55, 56]. The differences in nature of each medical school along with gender equality in medical education is an issue that should be addressed since it would affect medical students’ well-being and dropout. We believe that the coming age of gender diversity would inevitably impact worldwide medical education and would be a topic worth studying in the near future.

The main strength of our study was the large number of medical students compared to other previous studies in Thailand. Also, the reported dropout was the exact rate, and mental health problems were precisely diagnosed by psychiatrists. We measured potential contributing factors both before and after entry to medical education. Apart from common and widely studied variables, including academic failure or admission program, we also examined mental health problems, which is an essential but underemphasized factor. The mean age of all students was under twenty years, which captures a younger age group than students in most studies due to government policy. Based on the Asian context of educational and sociocultural factors, our study could enrich the comprehensive medical education research for the entire globe.

Nonetheless, some limitations of this study should be mentioned. The low number of dropouts may indicate the need for a longer follow-up beyond the preclinical period. Additional factors in terms of protective and probable risk factors, such as parental education, birth order, financial status, family problems, medical illness, and students’ motivation were not collected. We used mental health data from a single faculty-based site, which had limited service hours; hence the reported data might be lower than the actual prevalence because of stigmatization avoidance and student’s inconvenience [44, 57]. The students categorized to failing examinations received at least one academic F grade, which may not truly represent the academic failure. However, one study reported a strong association between dropout and lower academic achievement in the first two years of medical school [37]. Our study was designed in a cross-sectional fashion in one medical school locating in Bangkok. Therefore, causality could not be explained due to limitation of the study design, and generalizability to other Thai medical schools might be limited, especially in the regional areas.

## Conclusions

Dropout from medical education is complex in etiology. From the univariate analysis, academic failure plays a role in dropout among Thai medical students, but with adequate assistance, they are able to attain their medical degree. After adjusted for other confounding variables, we report that only having mental health problems is associated with dropout in this Thai medical school. Further longitudinal studies are needed to investigate protective and risk factors in higher clinical years or even in postgraduate medical education.

## Data Availability

The data that support the findings of this study are available from the Division of Academic Affairs, Faculty of Medicine, Chulalongkorn University, Bangkok, Thailand, but restrictions apply to the availability of these data, which were used under license for the current study, and so are not publicly available.

## Abbreviations

COTMES: The Consortium of Thai Medical Schools
GPA: Grade point average
